# Systemic immune-inflammation index combined with blood urea nitrogen to albumin ratio predicts 28-day mortality in sepsis: a retrospective cohort study

**DOI:** 10.3389/fphys.2026.1814729

**Published:** 2026-05-12

**Authors:** Ping Gong, Xuelian Yin, Xuanyu Hou, Zhen Zhou, Ziren Tang, Hong Zhao

**Affiliations:** 1 Department of Emergency Medicine, Shenzhen People’s Hospital (The Second Clinical Medical College, Jinan University; The First Affiliated Hospital, Southern University of Science and Technology), Shenzhen, Guangdong, China; 2 Department of Emergency Medicine, Beijing Tongren Hospital, Capital Medical University, Beijing, China; 3 School of Innovation and Development, Central University of Finance and Economics, Beijing, China; 4 School of Biomedical Engineering, Capital Medical University, Beijing, China; 5 Department of Emergency Medicine, Beijing Chao-Yang Hospital Affiliated to Capital Medical University, Beijing, China

**Keywords:** blood urea nitrogen to albumin ratio, mortality, predictive model, prognosis, sepsis, systemic immune-inflammation index

## Abstract

**Background:**

Sepsis remains a leading cause of mortality in intensive care units, highlighting an urgent need for simple, early prognostic biomarkers to improve risk stratification. The Systemic Immune-Inflammation Index (SII) and the Blood Urea Nitrogen to Albumin Ratio (BAR) reflect distinct pathophysiological axes—inflammatory-immune-coagulation and renal-metabolic dysfunction, respectively. However, their combined prognostic value in sepsis has not been explored.

**Methods:**

This single-center retrospective cohort study included older patients with sepsis (median age 82 years) admitted between 2022 and 2024. SII and BAR were calculated from admission laboratory data. The primary outcome was 28-day all-cause mortality. Predictive performance was assessed using ROC analysis and multivariable logistic regression, with internal validation via bootstrapping. A nomogram and a two-dimensional risk heatmap were developed to facilitate clinical application.

**Results:**

For predicting 28-day mortality, BAR (AUC: 0.721 (95% CI: 0.671–0.771)) outperformed SII (AUC: 0.669, 95% CI: 0.610–0.726). High SII (≥1072) and high BAR (≥8.64) were independent risk factors for mortality (OR: 3.613 and 4.471, respectively). The combined SII-BAR model demonstrated superior discrimination (AUC: 0.750, 95% CI: 0.713–0.822) and excellent calibration (Brier score: 0.110). Decision curve analysis confirmed a positive net benefit across clinically relevant threshold probabilities (10–30%). The nomogram and heatmap provided intuitive, individualized risk estimates.

**Conclusion:**

SII and BAR are independent and complementary predictors of 28-day mortality in sepsis. Their integration into a simple combined model significantly improves prognostic accuracy by capturing both inflammatory-immune and renal-metabolic dysregulation. These readily available biomarkers, together with the proposed visual tools, may facilitate early risk stratification and support personalized management in sepsis.

## Introduction

Sepsis, defined as life-threatening organ dysfunction caused by a dysregulated host response to infection, remains a leading cause of mortality in intensive care units (ICU) worldwide (Singer et al., 2016). Despite significant advancements in management, including the implementation of bundle strategies from the Surviving Sepsis Campaign Guidelines, mortality rates among septic patients continue to range from 20% to 40% (Singer et al., 2016; Evans et al., 2021). The complex pathophysiology of sepsis—characterized by concurrent hyperinflammation, immunosuppression, metabolic dysregulation, and coagulopathy—contributes to substantial heterogeneity in patient outcomes (Rhee et al., 2019). Consequently, there is an urgent need for simple, accurate, and readily available biomarkers to enable early risk stratification and guide personalized therapeutic interventions.

Currently, comprehensive scoring systems such as the Acute Physiology and Chronic Health Evaluation II (APACHE II) and the Sequential Organ Failure Assessment (SOFA) are widely employed to assess disease severity in clinical practice (Ho, 2007; Kaymak et al., 2018; Jiang et al., 2025; Liu et al., 2025). However, their utility in rapid decision-making is limited by the requirement for numerous clinical and laboratory parameters, complex calculations, and potential subjectivity in interpretation. In recent years, routine blood count-derived inflammatory indices—particularly the neutrophil-to-lymphocyte ratio (NLR) and platelet-to-lymphocyte ratio (PLR)—have gained attention due to their accessibility and reproducibility (Cai et al., 2024; Sharifi et al., 2026; Shen et al., 2026). Nevertheless, their predictive accuracy for sepsis outcomes remains suboptimal (Font et al., 2020).

The systemic immune-inflammation index (SII), a novel composite biomarker integrating neutrophil, lymphocyte, and platelet counts, offers a more comprehensive reflection of systemic inflammation, immune status, and coagulation activation (Li et al., 2024). Accumulating evidence has demonstrated the prognostic value of SII across a spectrum of diseases, including infections (Chen et al., 2025; Terzi et al., 2025), malignancies (Hu et al., 2014; Ohanoglu Cetinel et al., 2025; Jiao et al., 2026), cardiovascular disorders (Dong et al., 2025; Du et al., 2025; Sun et al., 2025; Ma et al., 2026), and other systemic conditions (Bai et al., 2025; Xu et al., 2025; Wang et al., 2026). Concurrently, the blood urea nitrogen to albumin ratio (BAR)—a simple index capturing both renal function (via urea nitrogen) and nutritional-inflammatory status (via albumin)—has emerged as a prognostic indicator in various acute illnesses, including sepsis (Min et al., 2022), pneumonia (Milas et al., 2022), COVID-19 (Hung et al., 2023), and other critical conditions (Yang et al., 2025; Li et al., 2026; Li et al., 2026; Wu et al., 2026). Notably, SII and BAR reflect distinct yet complementary pathophysiological dimensions of sepsis: SII primarily captures the “inflammation-immunity-coagulation” axis, while BAR represents the “renal function-nutrition-metabolism” axis.

Despite the biological plausibility of their synergistic application, research investigating the combined prognostic value of SII and BAR in sepsis remains scarce. To date, no study has systematically evaluated whether integrating these two accessible biomarkers can enhance mortality prediction in septic patients.

Therefore, this study aimed to: evaluate the independent and combined predictive value of admission SII and BAR for 28-day mortality in sepsis patients through a retrospective cohort analysis, and develop a visual clinical prediction model—including a nomogram and risk heatmap—to facilitate early risk stratification and support personalized clinical decision-making in sepsis management.

## Materials and methods

### Study population and design

This single-center retrospective cohort study consecutively screened patients diagnosed with sepsis according to the Sepsis-3 criteria (Singer et al., 2016) and admitted to the Emergency or General ICU of Beijing Tongren Hospital, Capital Medical University, between January 1, 2022, and December 31, 2024. The study protocol was approved by the local Ethics Committee (Approval No. PJ-2023-161), and the requirement for informed consent was waived given its retrospective design. All procedures complied with the Declaration of Helsinki, with strict protection of patient privacy.

### Participants

Patients were eligible if they: 1) were aged ≥18 years; 2) met the Sepsis-3 criteria for sepsis or septic shock (Singer et al., 2016); and 3) had an ICU length of stay >24 hours. Exclusion criteria were: 1) active malignancy; 2) pregnancy or lactation; 3) pre-existing severe chronic liver (e.g., Child-Pugh class C) or renal dysfunction (end-stage renal disease on dialysis) or history of solid organ transplantation; 4) primary hematologic or autoimmune diseases; 5) long-term use (>1 month) of corticosteroids or immunosuppressants; 6) receipt of blood product transfusion within one week prior to admission; 7) decision for palliative care or withdrawal of active treatment upon ICU admission; and 8) incomplete clinical or laboratory data required for analysis.

### Data collection and variables

Trained researchers extracted data from the hospital’s electronic medical record system using a standardized case report form. Collected variables included: Baseline and clinical characteristics: age, sex, body mass index (BMI), age-adjusted Charlson Comorbidity Index (ACCI), and primary site of infection; Severity of illness scores: The worst values within the first 24 hours of ICU admission were used to calculate the APACHE II and SOFA scores; Laboratory parameters: The first routine blood tests and biochemical profiles after ICU admission were recorded, including complete blood count (neutrophil, lymphocyte, platelet, monocyte, eosinophil counts), blood urea nitrogen (BUN), serum albumin, creatinine, and procalcitonin (PCT).

### Predictors and outcomes

The primary predictors were the SII and BAR, calculated as follows: SII: (platelet count × neutrophil count)/lymphocyte count; BAR (mg/g): (blood urea nitrogen × 28)/serum albumin, where blood urea nitrogen is in mmol/L and serum albumin is in g/L. The primary outcome was 28-day all-cause mortality from the time of ICU admission.

### Statistical analysis

All analyses were performed using R software (v4.3.0). Continuous variables were presented as median with interquartile range (IQR) and categorical variables as frequency (percentage). Univariable and multivariable logistic regression were used to identify factors associated with 28-day mortality, with results expressed as odds ratios (OR) and 95% confidence intervals (CI).

Discriminative ability was assessed using receiver operating characteristic (ROC) curve analysis, and the area under the curve (AUC) was calculated to evaluate predictive performance. Optimal cut-off values for SII and BAR were determined by the Youden index. Internal validation was conducted via bootstrapping with 1000 replicates. Calibration was evaluated using calibration plots and the Brier score, while clinical utility was assessed by decision curve analysis (DCA). Nonlinear associations were explored using restricted cubic splines (RCS). Collinearity between SII and BAR was examined using Spearman’s correlation and variance inflation factors (VIF). A nomogram and a two-dimensional risk heatmap were created for visualization of the combined model. Risk stratification was performed based on the optimal cut-offs, with group comparisons made by the chi-square test. A two-tailed *P*-value < 0.05 was considered statistically significant.

## Results

### Study population and baseline characteristics

A total of 729 patients with sepsis were included in the final analysis. Among the 93 patients with septic shock, 42 survived and 51 died. The cohort was predominantly elderly and critically ill, with a median age of 82 years (IQR: 70–88) and a median APACHE II score of 19 (IQR: 16–22). The key predictors of interest, the SII and BAR, exhibited substantial inter-patient variability, with median values of 1142.1 (IQR: 619–2218) and 6.93 (IQR:4.28, 11.95), respectively. Detailed baseline characteristics stratified by 28-day mortality status are presented in **Table 1**. Compared with survivors, non-survivors were significantly older, had higher comorbidity burden and severity scores, and displayed marked differences in inflammatory and metabolic markers, including elevated SII and BAR (all *P* < 0.001).

**Table 1:** Baseline characteristics of the study population.

Characteristic	Survivors (*n* = 630)	Non-survivors (*n* = 99)	Statistic	*P*-value
Male, *n* (%)	339 (53.8)	54 (54.5)	*χ*² = 0.001	0.978
Age, years	81.5 (68.0–87.0)	86.0 (80.0–91.0)	*Z* = –5.228	<0.001
Septic shock, *n* (%)	42 (6.7)	51 (51.5)	*χ² =* 154.8	<0.001
ACCI	8.0 (6.0–9.0)	9.0 (8.0–11.0)	*Z* = –5.148	<0.001
APACHE II score	18.0 (16.0–21.0)	21.0 (20.0–24.0)	*Z* = –8.010	<0.001
SOFA score	3.0 (2.0–4.0)	4.0 (3.0–5.0)	*Z* = –5.586	<0.001
ICU length of stay, days	15.0 (10.0–24.0)	12.0 (7.0–16.5)	*Z* = 4.338	<0.001
WBC, ×10^9^/L	7.70 (5.60–10.43)	9.64 (7.06–14.36)	*Z* = –4.438	<0.001
Neutrophil, %	76.8 (66.8–83.9)	85.6 (79.1–90.9)	*Z* = –7.268	<0.001
Lymphocyte, ×10^9^/L	1.02 (0.73–1.43)	0.73 (0.52–1.04)	*Z* = 5.758	<0.001
Platelet, ×10^9^/L	195.0 (145.0–271.0)	167.0 (127.5–229.0)	*Z* = 2.826	0.005
Eosinophil, ×10^9^/L	0.90 (0.20–2.40)	0.20 (0.00–0.79)	*Z* = 5.673	<0.001
Monocyte, ×10^9^/L	0.50 (0.35–0.72)	0.53 (0.31–0.75)	*Z* = –0.329	0.742
SII	1036 (577–1936)	1880 (1112–3491)	*Z* = –5.404	<0.001
BAR (mg/g) †	6.45 (4.02, 10.86)	11.95 (6.92, 18.86)	*Z* = –7.076	<0.001
Blood urea nitrogen, mmol/L	6.90 (4.40–11.00)	11.30 (6.55–18.85)	*Z* = –6.346	<0.001
Serum albumin, g/L	30.2 (26.0–35.0)	27.4 (23.0–32.0)	*t* = 5.046	<0.001

Data are presented as median (IQR) or *n* (%). † BAR was calculated as (blood urea nitrogen × 28)/serum albumin, where blood urea nitrogen is in mmol/L and serum albumin is in g/L. The multiplication factor 28 converts mmol/L of urea nitrogen to mg/L, yielding a ratio expressed as mg/g. ACCI, age-adjusted Charlson Comorbidity Index; APACHE II, Acute Physiology and Chronic Health Evaluation II; BAR, blood urea nitrogen to albumin ratio; ICU, intensive care unit; SII, systemic immune-inflammation index; SOFA, Sequential Organ Failure Assessment; WBC, white blood cell count.

### Predictive performance and risk stratification of SII and BAR

**Figure 1:**
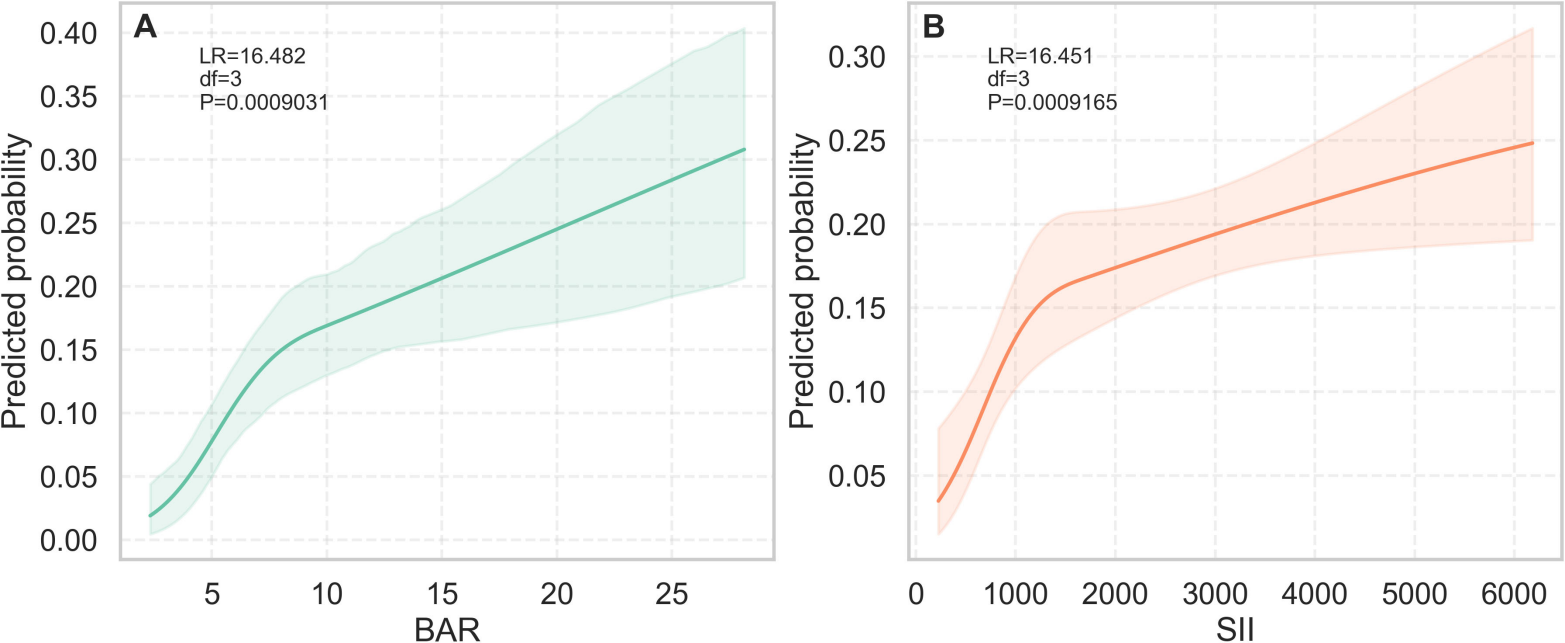
Distribution and diagnostic performance of BAR and SII. **(A)** Density plots showing the distribution of log-transformed BAR and SII; **(B)** ROC, Receiver operating characteristic curves of BAR and SII for predicting 28-day mortality. AUC, area under the curve; BAR, blood urea nitrogen to albumin ratio; CI, confidence intervals; SII, systemic immune-inflammation index.

The predictive value of SII and BAR for 28-day mortality was systematically evaluated. The distributions of both biomarkers, after log-transformation to account for their markedly different raw ranges, are shown as density plots in **Figure 1A**. Both exhibited right-skewed, unimodal profiles. SII showed a broader spread with a heavier upper tail, indicating greater inter-patient variability, whereas BAR was more tightly clustered with narrower dispersion.

Discriminative ability was assessed using ROC curve analysis (**Figure 1B**). BAR yielded an AUC of 0.721 (95% CI: 0.671–0.771) for predicting 28-day mortality, outperforming SII (AUC = 0.669, 95% CI: 0.610–0.726). The optimal cut-off values determined by the Youden index were 1072 for SII and 8.64 for BAR. Using these thresholds, patients were stratified into high- and low-risk groups. Both indices demonstrated significant risk stratification: the mortality rate in the high-SII group (19.9%) was 3.1-fold higher than in the low-SII group (6.4%). The high-BAR group exhibited an even greater mortality rate (24.4%), 3.6-fold higher than the low-BAR group (6.7%), suggesting that BAR may be superior for identifying the highest-risk patients.

After adjustment for age in multivariable logistic regression, both high SII (≥1072) and high BAR (≥8.64) remained independent risk factors for 28-day mortality. The association was slightly stronger for BAR (OR = 4.471, 95% CI: 2.852–7.164) than for SII (OR = 3.613, 95% CI: 2.231–6.077).

### Non-linear association and dose-response analysis

**Figure 2:**
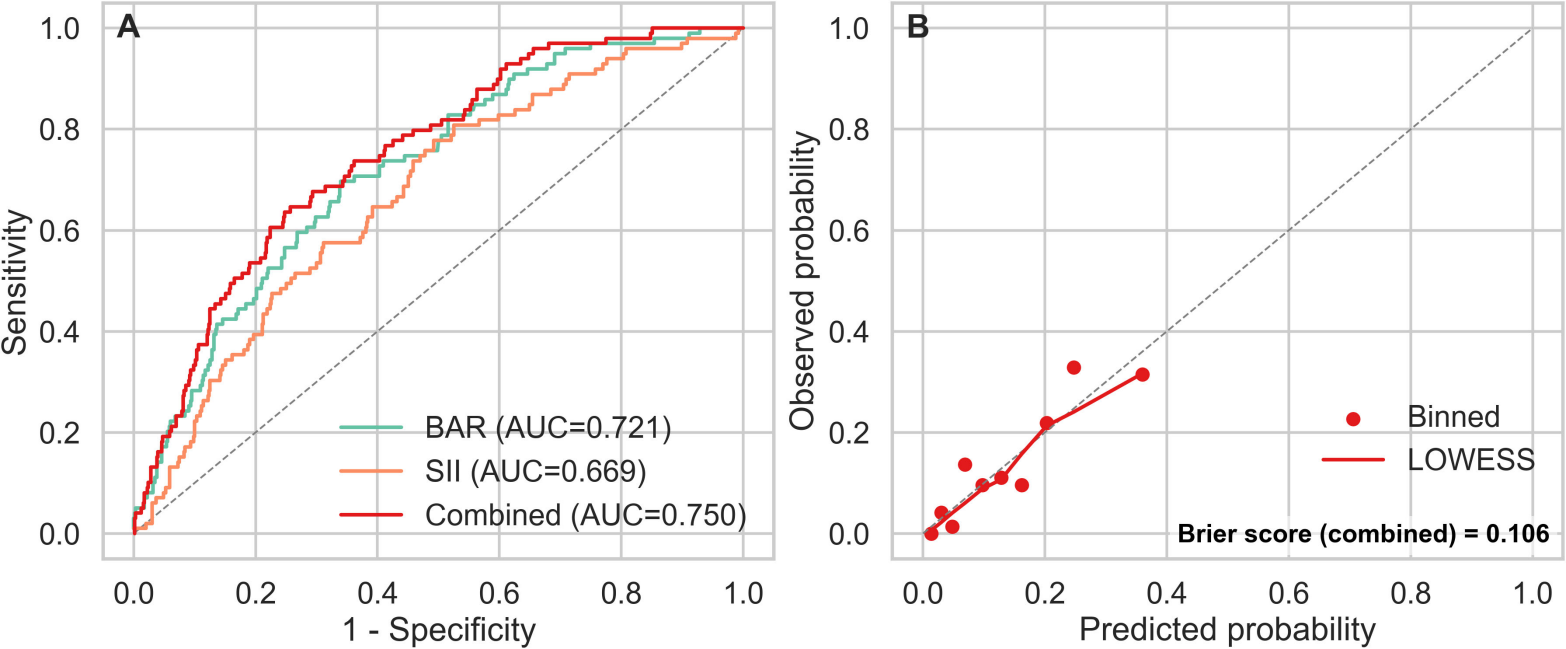
Dose-response relationships of BAR and SII with 28-day mortality. **(A)** RCS, Restricted cubic spline analysis of the association between BAR, blood urea nitrogen to albumin ratio and the predicted probability of 28-day mortality; **(B)** RCS analysis of the association between SII, systemic immune-inflammation index and 28-day mortality. Solid lines represent the predicted probability of death; shaded regions indicate 95% confidence intervals. *P* values for non-linearity were derived from likelihood ratio tests.

RCS analysis revealed significant non-linear relationships between both biomarkers and 28-day mortality (SII: likelihood ratio test = 16.451, df = 3, *P* < 0.001; BAR: likelihood ratio test = 16.482, df = 3, *P* < 0.001; **Figure 2**). As shown in **Figure 2A**, BAR demonstrated a monotonically increasing and approximately linear association with mortality risk; the probability of death rose progressively with higher BAR levels without evidence of a plateau, although the likelihood ratio test indicated a statistically significant departure from strict linearity. In contrast, **Figure 2B** illustrated that the association between SII and mortality exhibited a J-shaped pattern: within the clinically common range (SII < 5000), the probability of death increased with higher SII levels, with a particularly pronounced rise observed after SII exceeded 2000. The curve beyond SII > 5000 should be interpreted with caution due to limited sample size in that extreme range.

### Independence of SII and BAR and combined model development

**Figure 3:**
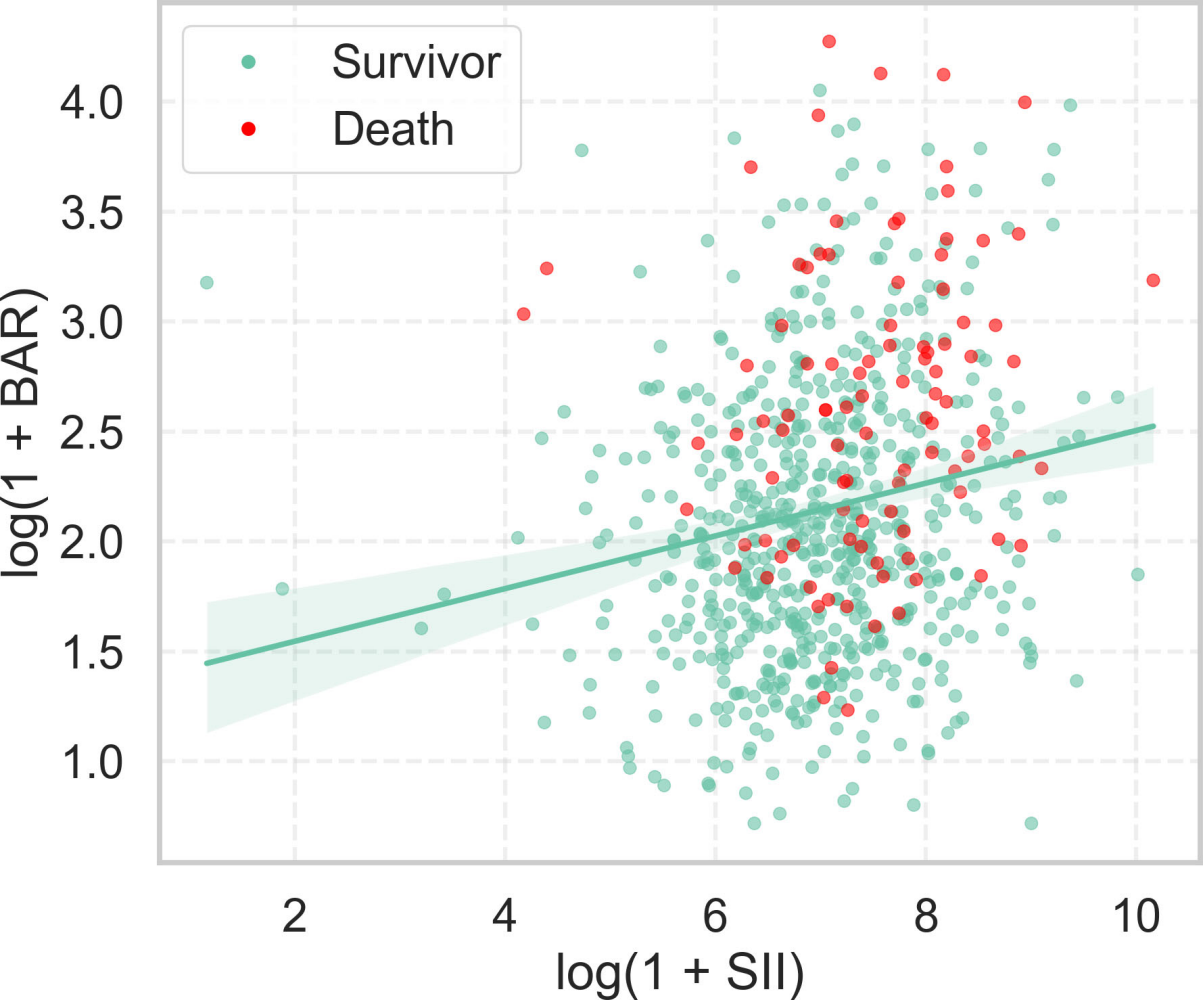
Correlation between BAR and SII. Scatter plot illustrating the relationship between log-transformed BAR and log-transformed SII. Green dots represent survivors; red dots represent non-survivors (28-day mortality). The green line indicates the linear trend with 95% confidence intervals. BAR, blood urea nitrogen to albumin ratio; SII, systemic immune-inflammation index.

Before constructing the combined model, we assessed the correlation and functional form between SII and BAR to justify their simultaneous inclusion. Spearman correlation analysis revealed a weak but statistically significant association (ρ = 0.197, *P* < 0.001; **Figure 3**), and collinearity diagnostics confirmed negligible multicollinearity (variance inflation factor [VIF] = 1.03).

To accommodate potential non-linear effects, RCS terms were specified for both predictors. We then evaluated whether a multiplicative interaction between the spline expansions of BAR and SII improved model fit. The interaction model did not yield a statistically significant improvement over the additive RCS model (likelihood ratio test: LR = 20.391, df = 16, *P* = 0.203), and the reduction in Akaike Information Criterion (AIC) was marginal (additive AIC = 523.178 vs. interaction AIC = 520.787). Accordingly, the final model was specified as an additive logistic regression with RCS for both BAR and SII: log [p/(1 - p)] = f_BAR (BAR; df=4) + f_SII (SII; df=4), where f_BAR and f_SII are smooth spline functions with 4 degrees of freedom each. This formulation allows the effect of each biomarker to vary flexibly across its observed range rather than assuming a constant slope, thereby balancing model flexibility with parsimony and mitigating overfitting risk while capturing the complementary prognostic information of BAR and SII. The interaction analysis was retained as a sensitivity check.

### Model validation and performance assessment

**Figure 4:**
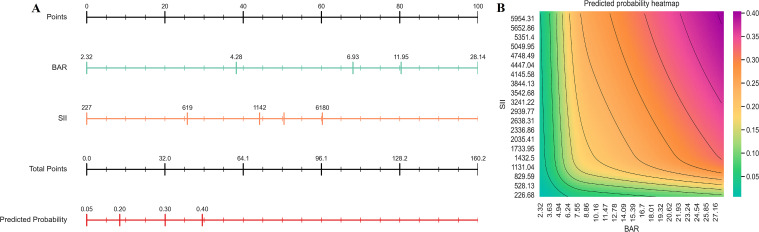
Validation of the combined model. **(A)** Bootstrap-validated ROC, receiver operating characteristic curve of the combined SII-BAR model for predicting 28-day mortality. The solid red line represents the model performance; the shaded area denotes the 95% confidence interval derived from 1000 bootstrap resamples; **(B)** Calibration plot comparing predicted versus observed probabilities of 28-day mortality. The ideal calibration is represented by the dashed diagonal line (y = x). The solid line indicates the actual calibration performance of the model.

The combined model incorporating both BAR and SII demonstrated good discriminative performance for predicting 28-day mortality. Using the RCS specification with four degrees of freedom, the combined model achieved an AUC of 0.750 (95% CI: 0.705–0.798), exceeding the performance of either marker alone (SII AUC = 0.669; BAR AUC = 0.721). Bootstrap resampling with 1000 iterations yielded a stable confidence interval for the combined AUC, supporting the reproducibility of its discriminative ability (**Figure 4A**).

Calibration assessment indicated close agreement between predicted probabilities and observed outcomes. The Brier score for the combined model was 0.106, and the calibration slope approximated 1.00, both consistent with well-calibrated predictions. Visually, the binned calibration points and LOWESS-smoothed curve tracked closely along the identity line (y = x) across the range of predicted probabilities (**Figure 4B**).

As reported in the previous section, testing for a multiplicative interaction between the spline expansions of BAR and SII did not significantly improve model fit (likelihood ratio test: LR = 20.391, df = 16, *P* = 0.203); therefore, the final model retained a parsimonious additive structure. Collectively, these results support modeling BAR and SII jointly with splines to capture their complementary, non-linear prognostic contributions while avoiding unnecessary complexity.

**Figure 5:**
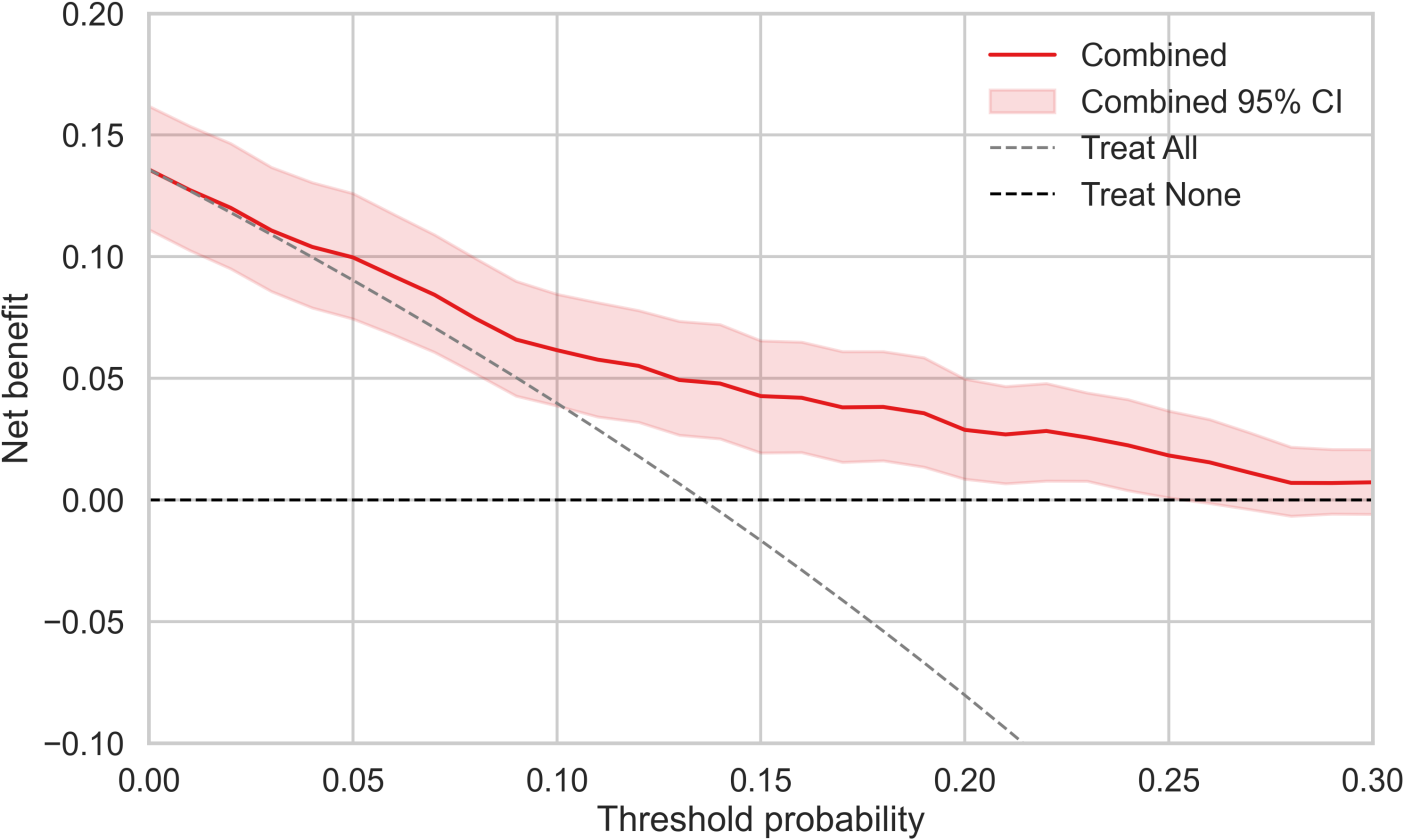
DCA, Decision curve analysis of the combined model. The graph illustrates the net benefit of the combined SII-BAR model (red line) compared with the “treat all” (gray dashed line) and “treat none” (black dashed line) strategies across a range of threshold probabilities for predicting 28-day mortality. The shaded area represents the 95% confidence interval derived from bootstrap resampling.

DCA demonstrated that the combined model (BAR + SII, RCS df = 4) yielded clinically meaningful net benefit across commonly used threshold probabilities. Within the 0.01–0.30 range, the combined curve consistently lay above the “treat none” strategy and outperformed the “treat all” strategy over most thresholds. For reference, at a threshold probability of 0.05, the net benefit of the combined model was approximately 0.10, compared with approximately 0.09 for the “treat all” strategy, reflecting practical clinical utility when the decision threshold is low to moderate. Bootstrap resampling with 1000 iterations provided a 95% confidence band around the combined curve, which remained predominantly above “treat none,” indicating robust and reproducible net benefit in this cohort (**Figure 5**).

**Figure 6:**
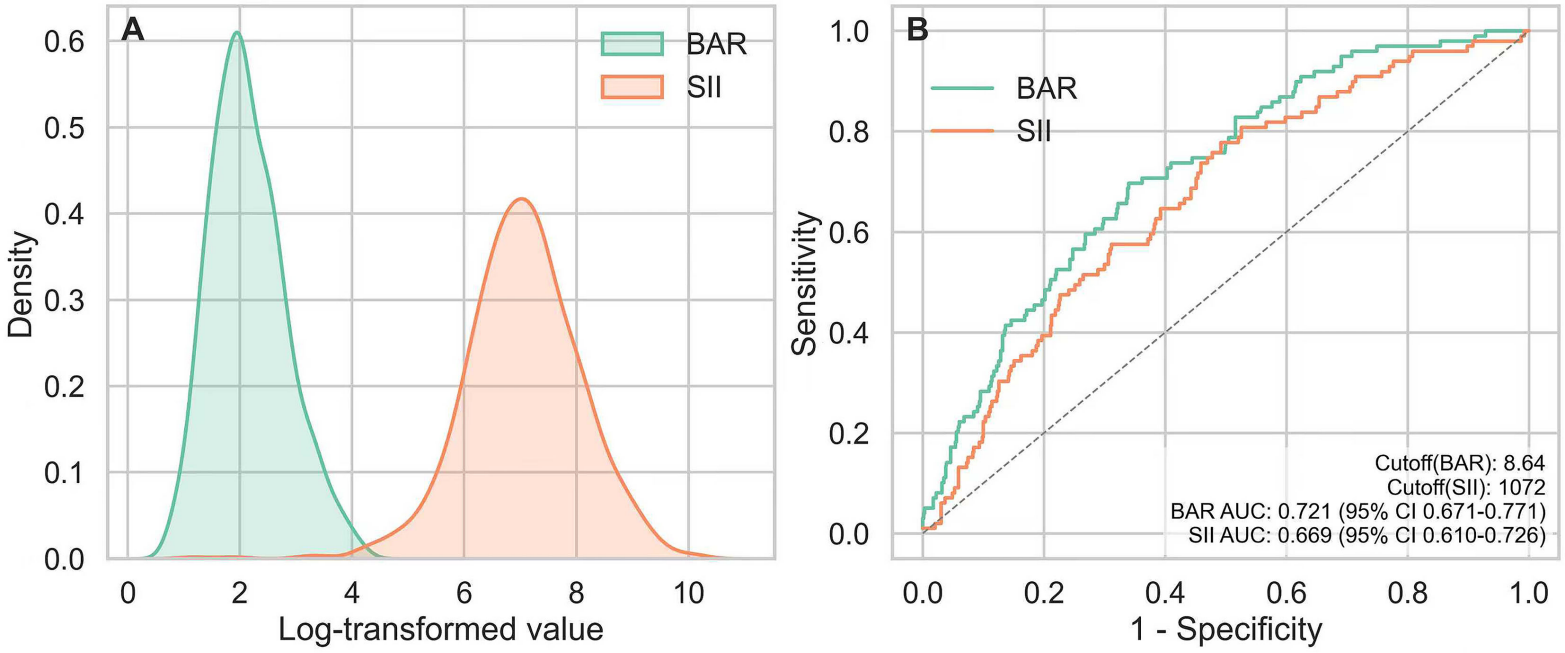
Visualization of the combined predictive model. **(A)** Nomogram for predicting the 28-day mortality risk. Each biomarker (BAR, SII) is assigned a score on the “Points” scale. The sum of these scores (“Total Points”) corresponds to a specific predicted probability of death; **(B)** Risk heatmap with contour lines illustrating the joint effect of BAR and SII on mortality risk. The color gradient represents the predicted probability of 28-day mortality; contour lines indicate specific probability thresholds.

A nomogram was constructed from the final additive logistic model using natural cubic splines for BAR and SII (df = 4) to facilitate clinical application (**Figure 6A**). The top axis provides a unified points scale; the BAR and SII axes map each predictor’s value to corresponding points via spline-based contributions. Total points are obtained by summing the points for BAR and SII, which are then translated to the predicted probability of 28-day mortality on the bottom risk axis. This format enables bedside estimation: the clinician locates the patient’s BAR and SII values on their respective axes, reads off the corresponding points, sums them, and converts the total points to an individualized risk estimate.

To visualize the joint effect of BAR and SII on 28-day mortality, a probability heatmap was generated from the same spline-based model (**Figure 6B**). The horizontal axis represents BAR and the vertical axis represents SII; colors depict the predicted probability using a cyan-to-purple-red gradient, and contour lines highlight regions of equal risk. Consistent with model estimates, the lower-left region (low BAR, low SII) corresponded to lower risk, while the upper-right region (high BAR, high SII) corresponded to higher risk, illustrating the complementary and non-linear contributions of both biomarkers to mortality risk.

## Discussion

Despite advances in sepsis management, it remains a leading cause of mortality among critically ill patients. This study directly addresses the persistent clinical need for simple, objective, and readily accessible biomarkers to facilitate early risk stratification—a gap underscored by the inherent limitations of existing multiparameter scoring systems in rapid bedside decision-making (Ho, 2007; Kaymak et al., 2018). Herein, we present the first systematic evaluation of the combined prognostic value of the SII and BAR for 28-day mortality in sepsis, offering a practical tool that uniquely integrates inflammatory-immune and renal-metabolic dimensions.

Our findings robustly confirm that both SII and BAR are independent predictors of 28-day mortality in sepsis, and their combination significantly enhances prognostic accuracy. Specifically: SII, reflecting systemic inflammation, immune status, and coagulation activation (Li et al., 2024), achieved an AUC of 0.669; BAR, indicative of renal function and metabolic-nutritional balance (Milas et al., 2022; Min et al., 2022), demonstrated superior discrimination with an AUC of 0.721; and their integration into a combined model yielded a substantially improved AUC of 0.768, underscoring their complementary prognostic value. This synergistic effect was further evidenced by the marked risk stratification capability—mortality rates in high-risk groups were 3.1-fold (SII) and 3.6-fold (BAR) higher than in low-risk groups. These findings answer the central question of whether two readily available indices, capturing distinct pathophysiological axes, could collectively improve predictive performance. To our knowledge, this is the first study to systematically develop and validate a combined SII-BAR model for mortality prediction in sepsis. While prior investigations have validated each marker individually in sepsis or other critical illnesses (Min et al., 2022; Ru and Luo, 2023; Wang et al., 2023), their synergistic application—integrating the “inflammation-immunity-coagulation” axis with the “renal function-nutrition-metabolism” axis—has not been previously explored, thereby providing a novel, physiologically coherent framework for multimodal risk assessment in sepsis.

Our results both align with and extend the existing literature. The independent prognostic value of SII and BAR is consistent with earlier reports (Min et al., 2022; Ru and Luo, 2023; Wang et al., 2023), reinforcing their utility as reliable biomarkers in sepsis. Notably, we observed a “J-shaped” relationship between SII and mortality risk, with a pronounced increase beyond an SII threshold of 2000. This nonlinear association echoes previous findings in sepsis cohorts (Ru and Luo, 2023) and may reflect a critical tipping point where excessive inflammatory and procoagulant responses become particularly lethal. In contrast, BAR exhibited a nearly linear dose-response relationship with mortality, supporting its role as a continuous marker of cumulative metabolic and renal stress. The weak correlation and low variance inflation factor between SII and BAR confirm their statistical independence, providing a robust justification for their combined use—a novel contribution to the field. This statistical independence, coupled with their distinct pathophysiological underpinnings, suggests that SII and BAR capture complementary aspects of sepsis progression, offering a more comprehensive prognostic picture than either marker alone.

The clinical implications of our findings are substantial. By leveraging routinely available laboratory parameters, the SII-BAR model circumvents the complexity and subjectivity inherent in traditional scoring systems, enabling rapid and objective risk assessment at the bedside. The derived nomogram and two-dimensional risk heatmap translate this model into intuitive visual tools that facilitate individualized risk estimation, aligning with the growing emphasis on precision medicine in sepsis care (Jiang et al., 2023). These tools empower clinicians to identify high-risk patients early, potentially guiding more timely and targeted interventions—such as intensified monitoring, early source control, or personalized immunomodulatory therapy—while avoiding unnecessary interventions in low-risk patients. This approach exemplifies how integrating complementary biomarkers can provide a more holistic view of sepsis pathophysiology, moving beyond unidimensional assessments toward a multimodal risk stratification paradigm.

However, several limitations warrant consideration. First, the single-center retrospective design may introduce selection bias and limit the generalizability of our findings to broader populations or different healthcare settings. Second, our analysis relied solely on admission values of SII and BAR; dynamic changes in these biomarkers during the course of treatment could offer additional prognostic insights and should be investigated in prospective longitudinal studies. Third, our cohort was characterized by a high median age (82 years) and relatively low SOFA scores (median 3–4), indicating predominantly mild to moderate disease severity. Therefore, our findings may not be directly generalizable to younger populations or to sepsis patients with higher SOFA scores and more severe organ dysfunction. External validation in cohorts encompassing a broader spectrum of illness severity is warranted. Fourth, our study included patients with both sepsis and septic shock. While we reported the proportion of septic shock patients (12.8%), the small subgroup size precluded a robust separate analysis of prognostic performance in septic shock alone. Future studies with larger septic shock cohorts are needed to validate our findings in this specific population. Fifth, while internal validation through bootstrapping demonstrated robust performance, external validation in large, multicenter, prospective cohorts is essential before this model can be recommended for routine clinical implementation. Finally, although we adjusted for key confounders, residual confounding from unmeasured variables cannot be entirely excluded given the observational study design.

In conclusion, this study demonstrates that SII and BAR are independent and complementary predictors of 28-day mortality in sepsis. Their integration into a simple combined model significantly enhances prognostic accuracy by capturing both inflammatory-immune and renal-metabolic dysregulation. The derived nomogram and risk heatmap offer practical, visually intuitive tools for early risk stratification at the bedside. Future multicenter prospective studies are warranted to validate these findings across diverse populations and to explore the utility of these biomarkers in guiding personalized sepsis management strategies.

## Data Availability

The raw data supporting the conclusions of this article will be made available by the authors, without undue reservation.
